# The Antiinfective Effects of Velvet Antler of Formosan Sambar Deer (*Cervus unicolor swinhoei*) on *Staphylococcus aureus*-Infected Mice

**DOI:** 10.1155/2011/534069

**Published:** 2011-04-07

**Authors:** Ting-Yeu Dai, Chih-Hua Wang, Kun-Nan Chen, I-Nung Huang, Wei-Sheng Hong, Sheng-Yao Wang, Yen-Po Chen, Ching-Yun Kuo, Ming-Ju Chen

**Affiliations:** ^1^Department of Animal Science and Technology, National Taiwan University, No. 50 Lane 155 Sec. 3 Keelung Road, Taipei 106, Taiwan; ^2^Kaohsiung Animal Propagation Station, Livestock Research Institute, Council of Agriculture, Kaohsiung, Taiwan; ^3^Department of Mechanical Engineering, Tungnan University, Taipei 222, Taiwan; ^4^Livestock Research Institute, Council of Agriculture, Tainan, Taiwan; ^5^Center for Biotechnology, National Taiwan University, No. 81, Changxing, Taipei 106, Taiwan

## Abstract

We assayed the effects of velvet antler (VA) of Formosan sambar deer (*Cervus unicolor swinhoei*) and its extracts on the anti-infective activity against pathogenic *Staphylococcus aureus in vitro* and *in vivo* in this study. *In vitro* data indicated that the VA extracts stimulated the proliferation of resting splenocytes and macrophages in a dose-dependent manner up to the highest concentration used (150 *μ*g mL^−1^). The production of proinflammatory cytokines (TNF-*α*, IL-6, IL-12) by lipoteichoic acid was significantly suppressed after being cocultured with the VA extracts in a dose-dependent manner. Animal test in *S. aureus*-infected mice demonstrated that the numbers of bacteria determined in the kidneys and peritoneal lavage fluid of *S. aureus*-infected mice were significantly higher than those found in the same organs of mice pretreated with the VA samples. Moreover, the highly enhanced phagocytic activity of macrophages was further verified after *in vitro* treatment with the VA samples. The protective mechanisms of the VA samples might include an immune enhancer and an inflammatory cytokine suppressor.

## 1. Introduction

Formosan sambar deer (*Cervus unicolor swinhoei*) is an indigenous subspecies in Taiwan [1]. The use of its antler velvet has also steadily increased since the start of deer farming in Taiwan in 1963. Velvet antler (VA), the unossified antler of Family Cervidae, has been used in traditional Chinese medicines and healthy food for over 2000 years [2]. There are many different carbohydrates, peptides, lipids, sterols, and inorganic substances (Ca, Zn, Pb) in VA, which contains many different carbohydrates, peptides, lipids, sterols, and inorganic substances (Ca, Zn, Pb) [3, 4], should be beneficial to health. Many reports and clinical observations convincingly show that VA and its extracts are composed of many compounds with different functional groups, such as epidermal growth factor [5], proteoglycans with a hyaluronic acid core [6], and some water soluble compounds (carbohydrates, hexosamines, hydronyprolines, mucopolysacchride, sialic acids, uronic acids) as well as water insoluble fatty acids (prostaglandins, phospholipids, glycolipids, and gangliosides) [4]. Those active compounds may be the reasons of inhibitory activities on arthritis [7, 8], immunomodulatory activities [9], antinarcotic effects [10], and inhibitory action on monoamine oxidase [2]. Recent studies have shown that polysaccharides and lysophosphatidylcholines are responsible for antiulcer and hypertensive actions, respectively [11]. It has also been reported that the phosphates and gangliosides of VA can ease the symptoms of senility [12, 13].

It is widely believed that VA and its extracts have immunomodulatory activities. It was reported that orally administrated VA extracts increased monocytes in rats [14]. Their increase may enhance immune function. Intraperitoneal injection of pantocrin enhanced phagocytosis and immunoglobulin levels in mice [15]. Suh et al. [16] examined ethanol extracts from antlers of *Cervus nippon*, which enhanced the phagocytic activity of murine peritoneal macrophages. Kim et al. [9] further identified the active compounds and suggested that phosphatidylcholines with saturated fatty acyl chains might be immunostimulating factors. In addition, VA extracts have been used in the prevention and treatment of certain immune-related diseases, such as rheumatoid arthritis. Since some researches provide evidences that velvet antler might effectively relieve inflammation *in vitro* and *in vivo* [17], VA might have the potential to be used as possible remedy to treat septic syndrome. However, there is a lack of evidence for antiinfective effects of VA and its extracts. The underlying mechanisms are also fairly unknown.

*Staphylococcus aureus* is a major human pathogen that causes various types of infections and disease syndromes. It is the most familiar Gram-positive pathogen that produces a wide array of toxins [18]. Infection with *S. aureus* may cause severe sepsis and lead to septic shock. It will be considered as an appropriate bacterium in infection model [19]. Thus, the purpose of this study was to evaluate the effects of VA of Formosan sambar deer and its extracts on the antiinfective activities against pathogenic *S. aureus in vitro* and *in vivo*.

## 2. Materials and Methods

### 2.1. VA Samples

VA from Formosan sambar deer was harvested in Kaohsiung Animal Propagation Station, Taiwan Live Stock Research Institute (Pintong, Taiwan) after a 75-day VA growing period. Fresh VA was immediately sliced and frozen at −20°C. The frozen VA slices were dehydrated by lyophilization (Freeze dryer, Kingmech Co. Ltd., Taipei, Taiwan) and then grinded into fine powder (VA powder) less than 100 mesh by pulverizing machine (No. RT-02A, Rong Tsong Co., Taichung, Taiwan). The VA cold-water-soaked extract (VACWS extract) was obtained by soaking the VA powder with cold distilled water (50 g L^−1^) and stirring with a magnetic stir bar at 4°C for 24 hrs. The VA water-boiled-extract (VAWB extract) was conducted by cooking the VA powder with boiling distilled water (50 g L^−1^) for 6 hrs. After the soaking and boiling process, the VA extracts were centrifuged (1000 × g, 10 min), and the insoluble components were discarded. The VA extracts were then freeze-dried and stored at −20°C. 

For *in vitro* assays, the dehydrated VA extracts were dissolved in Dulbecco's phosphate buffered saline (DPBS; Thermo Fisher Scientific Inc., Logan, UT, U.S.A.) and sterilized by passing through 0.22 *μ*m filters (Millipore Corp., Carrigtwohill, Ireland). The extract samples were dissolved and diluted immediately before the assays were performed.

### 2.2. Experimental Animals

Six-week-old female BALB/cByJNarl mice (National Laboratory Animal Center; NLAC, Taipei, Taiwan) were accustomed to their new environment for at least 1 week before the start of the experiment. The mice were maintained in an automatic light/dark cycle (light periods of 14 hrs). Temperature and humidity were kept constant at 22°C and 50%, respectively. The animal care and treatment were performed in accordance with the guidelines of the National Science Council of Republic of China.

### 2.3. Cell Cultures

The murine macrophage cell line RAW 264.7 (ATCC TIB-71), obtained from Bioresource Collection, and Research Center (BCRC, HsinChu, Taiwan), was cultured in a complete Dulbecco's Modified Eagle's Medium (DMEM; Thermo) supplied with 10% heat-inactivated fetal bovine serum (hFBS; Invitrogen, Carlsbad, CA, U.S.A.) and 1% antibiotics (Antibiotic-Antimycotic; Invitrogen). The cells were maintained in 95% air with 5% CO_2_ at 37°C in a humidified incubator (Revco, Santa Fe Springs, CA., U.S.A.).

For splenocytes, the mice were sacrificed by cervical dislocation and splenocytes were harvested for culture. The preparation of splenocyte cultures was based on the procedure as described by Zhao et al. [20]. Murine peritoneal cells were isolated from seven-week-old female BALB/cByJNarl mice (NLAC) as previously described by Zhang et al. [21] and Hong et al. [22]. Cell number and viability were enumerated by 0.4% trypan blue stain (Invitrogen) exclusion on a hemacytometer (Superior, Marienfeld, Germany) using an inverted microscope (Olympus Optical Co. Ltd., Tokyo, Japan).

### 2.4. Pathogenic *S. aureus*

Pathogenic *S. aureus* subsp. *aureus* Rosenbach (BCRC12651) was propagated in tryptic soy broth (BD DifcoTM) and incubated at 37°C. The bacterial counts of *S. aureus* were determined by a 10-fold series dilution with 0.85% saline, and 0.1 mL of dilution were cultured on blood agar plate (Creative Media Products, Ltd., Taipei, Taiwan) at 37°C for 24 hrs.

### 2.5. *S. aureus*-Infected Mice Model

Specific pathogen-free female 6-week-old BALB/cByJNarl mice (NLAC) were divided into groups (*n* = 5–8), each of which were intragastrically (i.g.) administrated with distilled water (200 *μ*L mouse^−1^), the VA extracts (200 *μ*L mouse^−1^), or the VA powder (0–500 mg kg^−1^ mouse body weight), respectively, each day for 28 days. The mice were then i.p. infected with 1.5 × 108 cfu mouse^−1^ live pathogenic *S. aureus* cells suspended in 200 *μ*L of DPBS.23. After 48-hr pathogenic injection, blood was collected by cheek punch with animal bleeding lancet (GoldenRod 5.0 mm point size; MEDIpoint Inc., Mineola, New York, U.S.A.) and Microtainer tubes (BD, Franklin Lakes, NJ., U.S.A.) containing clot activator and polymer gel. The infected mice were then sacrificed by cervical dislocation and injected 10 mL of cold DPBS into the peritoneal cavity. Peritoneal lavage fluid (PLF), kidneys and spleen of infected mice were also collected for further measurement of organs' weight and bacterial counts. The blood was centrifuged (25°C, 10,000 × g, 5 min). The serum was separated and stored at −80°C for cytokine assays.

### 2.6. Cell Proliferation Assay

RAW 264.7 cells and murine splenocytes were cultured in sterile 96-well flat-bottomed plates (TPP, Trasadingen, Switzerland) (5 × 10^3^ cells well^−1^) with or without the VA extracts (25 to 150 *μ*g mL^−1^) and incubated at 37°C for 12 hrs. Ten *μ*g mL^−1^ of lipoteichoic acid (LTA; Sigma, St. Louis, MN., U.S.A.) was used as a positive control. Cell proliferation assay was performed by BrdU Cell Proliferation kit (Colorimetric; Roche, Mannheim, Germany) according to manufacturer's instruction.

### 2.7. Cytokine Assays

RAW 264.7 cells and the murine peritoneal cells were plated to 24-well flat-bottomed plate (BD FalconTM, Franklin Lakes, NJ, U.S.A.) (2 × 10^5^ cells well^−1^) and cocultured in the VA extracts (25 to 200 *μ*g mL^−1^) with or without 10 *μ*g mL^−1^ LTA at 37°C for 24 hrs. The cytokine levels of murine tumor necrosis factor (TNF)-*α*, interleukin (IL)-6 and IL-12 p40 in cell supernatant and IL-6 and transforming growth factor (TGF)-*β*1 in mice sera were measured by Duoset sandwich ELISA kit (R&D Systems, Minneapolis, MN, U.S.A.) according to manufacturer's instruction. The results were expressed as the concentration of each cytokine.

### 2.8. Phagocytosis Assay

Fluorescein-isothiocyanate-(FITC)-labeled *S. aureus* cells were prepared as described by Gov et al. [18]. RAW 264.7 cells were pretreated with 100 *μ*g mL^−1^ of the VA extracts for 6 hrs at 37°C before the phagocytic assay was performed. The phagocytic activities were determined as described by Shi et al. [23]. Briefly, macrophages (5 × 10^6^ cells) were coincubated with opsonized FITC-*S. aureus* (5 × 10^8^ cells) in 1 mL DPBS at 37°C for 30 min. The macrophage cells were then washed 4 times with DPBS to eliminate the extracellular bacteria. The ingested FITC-*S. aureus* was analyzed by Cytomic FC500 flow cytometer (Beckman Coulter, Fullerton, CA, U.S.A.).

### 2.9. Statistical Analysis

Differences between groups were compared using SAS software package version 9.1 (SAS Institute Inc., Cary, NC, U.S.A.). Statistical comparisons were analyzed by one-way analysis of variance (ANOVA), followed by Duncan's multiple-range test to locate differences. The results were presented as mean ± standard error of mean (S.E.M.).

## 3. Results

### 3.1. Effect of VA Extracts on Cell Proliferation In Vitro

**Figure 1:**
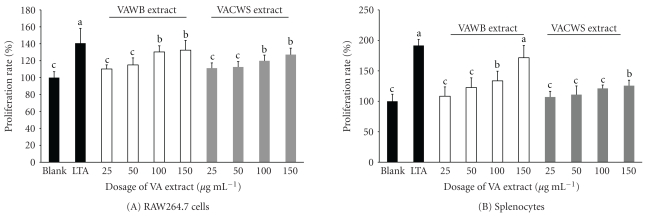
The VA extracts enhance cell proliferation activities in (A) RAW 264.7 cells; (B) splenocyte. Proliferation rate were calculated by the absorbance value of blank group as 100%. Each experimental value was divided with the absorbance value of blank group. Values are mean ± S.E.M. Asterisk denotes values that are significantly different from positive control group (LTA group). (a–c) Means within the same column without the same superscripts differ significantly (*P* < .05, *n* = 5).

The effects of the VA extracts on murine macrophage and splenocyte proliferation were evaluated (Figure 1). The VAWB extract stimulated the proliferation of resting splenocytes and macrophages in a dose-dependent manner up to the highest concentration used (150 *μ*g mL^−1^). The stimulating effect became significant at cocultured with 50 *μ*g mL^−1^ of the VAWB extract for both cells when compared with blank as control. At concentration of 150 *μ*g mL^−1^, the proliferation rate of murine macrophages and splenocytes for the VAWB extract were 171.5% and 132.4%, respectively. Similar results were also found in the VACWS extracts. The VACWS extracts (100–150 *μ*g mL^−1^) induced the proliferation of splenocytes and macrophages in a dose-dependent manner. At concentration of 150 *μ*g mL^−1^, the proliferation rates of murine macrophages and splenocytes for the VACWS extracts were 127.2% and 125.7%, respectively.

### 3.2. Effect of VA Extracts on Cytokine Inhibition In Vitro

**Figure 2:**
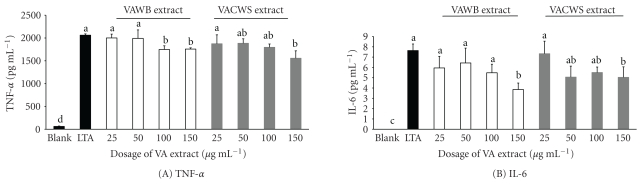
Inhibition of cytokine (A) TNF-alpha; (B) IL-6 secretion in RAW264.7 cells. Cells were stimulated with LTA (10 *μ*g/mL) under the treatment of water boiled (VAWB) extract and cold water soaked (VACWS) extract within the range of 25 *μ*g mL^−1^ to 200 *μ*g mL^−1^. Values are mean ± S.E.M. Asterisk denotes values that are significantly different from positive control group (LTA group). (a–c) Means within the same column without the same superscripts differ significantly (*P* < .05, *n* = 4).

**Figure 3:**
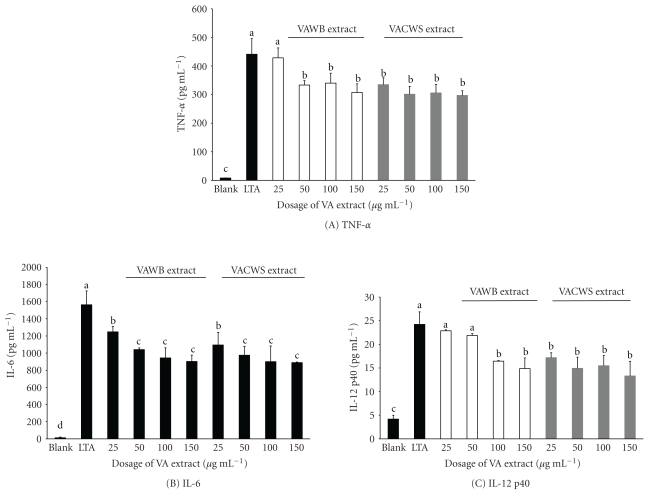
Inhibition of cytokine (A) TNF-alpha; (B) IL-6; (C) IL-12 p40 secretion in peritoneal cells. Cells were stimulated with LTA (10 *μ*g mL^−1^) under the treatment of water-boiled (VAWB) extract and cold water-soaked (VACWS) extract within the range of 25 *μ*g mL^−1^ to 150 *μ*g mL^−1^. Values are mean ± S.E.M. Asterisk denotes values that are significantly different from positive control group (LTA group). (a–c) Means within the same column without the same superscripts differ significantly (*P* < .05, *n* = 4).

The effects of the VA extracts on cytokine inhibition by murine macrophages in the presence of LTA are shown in Figure 2. The production of proinflammatory cytokines (TNF-*α*, IL-6) from the macrophages by adding LTA was significantly suppressed after being cocultured with the VA extracts in a dose-dependent manner up to the highest concentration used (150 *μ*g mL^−1^). The inhibition effects of TNF-*α*, IL-6, and IL-12 became significant in microphages cocultured with 100 *μ*g mL^−1^ of either VABW or VACWS extracts when compared with LTA. Similar results were also found in peritoneal cells (Figure 3). The production of proinflammatory cytokines (TNF-*α*, IL-6, IL-12 p40) from peritoneal cells by adding LTA was significantly suppressed after being cocultured with both VA extracts in a dose-dependent manner.

### 3.3. Effect of VA Extracts on Phagocytic Activity In Vitro

**Figure 4:**
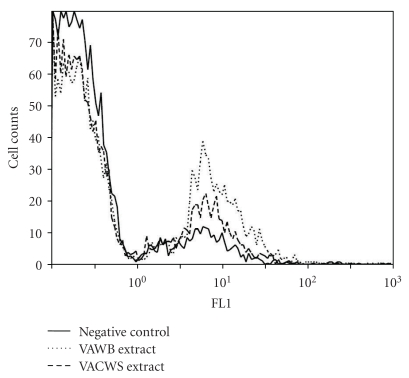
The VA extract enhance phagocytosis activities in heat-killed *S. aureus*-infected macrophages. Bold line: macrophages without pretreatment, dotted line: macrophages pre-treated with the VA water-boiled (VAWB) extract, dashed line: macrophages pre-treated with the VA cold water soaked (VACWS) extract. A representative of three separate assays is shown.

The phagocytic activity of macrophages was investigated to confirm if it increased with the presence of the VA extracts. Microphages treated by the VA extracts (Figure 4) demonstrated an enhanced phagocytic acidity against *S. aureus* when compared to the nontreated control macrophages. In particular, the VAWB extract-treated macrophages demonstrated 2.6-fold increases in phagocytic activity. These findings indicated that the VA extracts excited macrophages and further accelerated the antibacterial and phagocytic activities of macrophages.

### 3.4. Effect of Oral Feeding of the VA Extracts and the VA Powder on *Staphylococcus aureus*-Infected Mice

#### 3.4.1. Inhibited *S. aureus* Activities by the VA Samples In Vivo

The numbers of *S. aureus* in PLF and kidneys of the mice treated by the VA samples (the VAWB extract, VACWS extract and the VA powder) were significantly lower than those of the control mice (Table 1). There were no significant differences among the different VA samples. Additionally, the effects of different dosages of the VA powder samples on *S. aureus* counts were studied (Table 2). Except for mice treated with 2.5 mg of the VA powder, the concentrations of *S. aureus* in PLF and kidneys of the VA sample-pretreated mice were significantly lower than those from the control mice. There were no significant differences among different dosages. These results demonstrated that pretreatment of mice with the VA samples decreased significantly *S. aureus* number in the kidney and the PLF.

The organ weights of *S. aureus*-infected mice administrated with the different VA samples were also evaluated. Infected kidneys in the mice pretreated with 5 and 10 mg of the VA powders weighed significantly less than those of the control mice (Tables 1 and 2), whereas infected kidneys in the VA extract-pretreated mice showed no significant difference with the control mice in weight.

#### 3.4.2. Reduced Inflammatory Cytokines by the VA Samples

**Figure 5:**
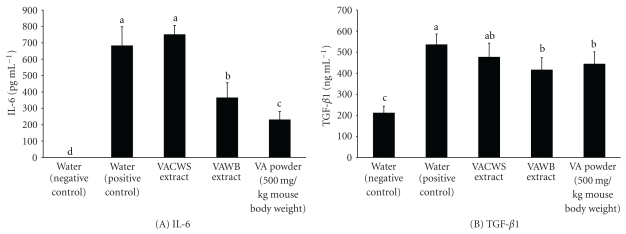
Cytokine concentration in serum of *S. aureus*-infected mice (A) IL-6; (B) TGF-*β*1. Values are mean ± S.E.M. (a–c) Means within the same column without the same superscripts differ significantly (*P* < .05, *n* = 5).

**Figure 6:**
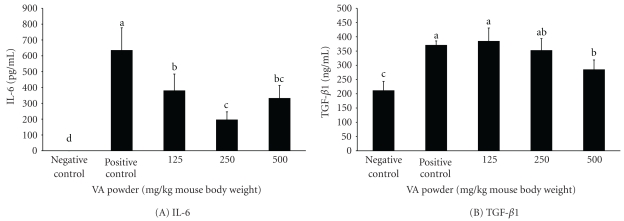
Cytokine concentration in serum of *S. aureus*-infected mice administrated with different dosages of the VA powders (A) IL-6; (B) TGF-*β*1. Values are mean ± S.E.M. (a–c) Means within the same column without the same superscripts differ significantly (*P* < .05, *n* = 8).

Blood samples from the VA sample-treated and subsequently *S. aureus*-infected mice and from the *S. aureus*-infected mice without pretreatment were both evaluated for levels of inflammatory cytokines in serum (Figure 5). The levels of IL-6 and TGF-*β*1 in serum were high for the mice infected with *S. aureus* without pretreatment. On the other hand, mice that were pretreated with the VAWB extract and the VA powder produced significantly lower levels of IL-6 and TGF-*β*1 than mice with no treatment. Interestingly, mice treated with the VACWS extract did not show any reduction in IL-6 and TGF-*β*1. The effects of different dosages of the VA powder on the production of inflammatory cytokines were also studied (Figure 6). Levels of IL-6 in serum were significantly downregulated for mice pretreated with all VA powder samples. The lowest levels of IL-6 were detected in the serum for the mice fed with 5 mg or higher of the VA powder. A decrease of TGF-*β*1 in serum was observed only when mice were treated with 10 mg of the VA powder.

Proinflammatory cytokines are known important mediators in development of lethal sepsis. Our data indicated that the VA samples significantly inhibited the production of proinflammatory cytokines, which might effectively decrease the septic symptoms of mice. 

## 4. Discussion

*Staphylococcus aureus* is a virulent pathogen that has the ability to cause a variety of potentially life-threatening infections. Previous reports on the correlation VA extracts against *S*. *aureus* infection are rare. In this study, we demonstrated that the VA samples stimulated the proliferation of resting splenocytes and macrophages in a dose-dependent manner (Figure 1). The spleen is an ovoid secondary lymphoid organ that plays a major role in mounting immune responses to antigens in the blood stream [24]. Splenocytes, obtained from spleen, refer to T-cells, B-cells, nature killer cells, mononuclear cells, macrophages, and monocytes, which are characterized by the expression of cell surface or other markers [9]. Macrophages represent 3–7% of leukocytes in blood and are necessary to the immune functions of lymph, spleen, and bone marrow [25]. Increasing macrophages enhance the immune functions. A couple of studies also reported that the bioactivity of the deer antler might act as an immune enhancer [16, 26].

Additionally, we confirmed that the VA extracts strongly inhibited the production of proinflammatory cytokine (Figures 2 and 3), such as TNF-*α*, IL-6, and IL-12 p40, in response to *in vitro* stimulation with bacterial LTA in a dose-dependent manner. These proinflammatory cytokines are produced by activated macrophages and other cell types. TNF-*α* is known to enhance the expression of accessory molecules involved in the adhesion of macrophages to the endothelium, increasing the possibility that other memory cells present in the circulatory system are guided to the inflammation site [27]. IL-12 is related to the differentiation of naïve T cells into T helper type 1 cells, which stimulates the production of TNF-*α* from T and natural killer cells. The naïve T cells' activation and proliferation create an acquired immune response to the newly encountered pathogenic agent [22]. IL-6 is multifunctional cytokine involved in diverse biological processes, such as the growth factor for normal or neoplastic cells [28], the host response to enteric pathogens, and terminal differentiation of B lymphocytes [29]. However, increased concentrations of proinflammatory cytokines may attenuate excessive host inflammatory responses. Suh et al. [17] reported that the abundance of TNF-*α* in the arthritic joints provides evidence of its involvement in the disease pathology. Neutralization of TNF-*α* leads to reduce production of other inflammatory cytokines [30]. In response to *in vitro* stimulation with bacterial LTA for the downregulation of proinflammatory cytokines, we assumed that the VA extracts were able to deliver immune signals to macrophages and other cells and might possess anti-inflammatory properties. 

Further animal tests on *S. aureus*-infected mice were also conducted to verify antiinfective effects of the VA samples. The mice that were treated with 5 mg or higher of the VA powder for 28 days before being challenged with *S*. *aureus* exhibited the best protection against sepsis. The numbers of *S*. *aureus* determined in the kidneys and PLF of *S. aureus*-infected mice were significantly higher than those found in the same organs of mice pretreated with the VA samples (Tables 2). Moreover, the highly enhanced phagocytic activity of macrophages (Figure 4) was further verified after *in vitro* treatment with the VA samples. Most phagocytosis is conducted by specialized cells, such as blood monocytes, neutrophils, and tissue macrophages. Monocyte/macrophage-mediated protection of animals was also demonstrated in *S. aureus*-induced endocarditis and sepsis models in different systems [25, 31]. Satorres et al. [32] indicated that phagocytic killing played an essential role to eliminate *S. aureus* in the host. The patients who were neutropenic or who have defects in polymorphonuclear leukocyte function suffered recurrent staphylococcal infections. 

Major inflammatory cytokines such as IL-6 and TGF-*β*1 in serum of *S. aureus*-infected mice were also studies. The excessive productions of IL-6 and TGF-*β*1 in serum were observed in *S. aureus*-infected mice not pretreated with the VA samples. Conversely, the levels of IL-6 and TGF-*β*1 in serum were suppressed for the mice pretreated with the VA samples (Figures 5 and 6). In accordance with our results, low levels of IL-6 and TGF-*β*1 in serum were also found in vaccinated mice [33]. Yao et al. [34] indicated that cytokine expression in the tissues correlated with leukocyte migration into the affected tissue and with evidence of progressive tissue damage. Early and intense production of IL-6 corresponded to a rapid worsening of articular lesions [35]. 

Furthermore, IL-6 and TGF-beta are recognized as T-helper 17 (Th17)-inducing cytokines. Th17 cytokines such as IL-17 have been associated with several conditions, including airway inflammation, rheumatoid arthritis, and inflammatory bowel disease [36]. IL-6 not only enhances production of Th17 [37], but also suppresses the expression of regulatory T cell (Treg) differentiation, favoring expression of the Th17 cells [38]. Although the role of TGF-*β* in Th17 development is unclear, a couple of studies have indicated that TGF-*β* is necessary for human Th17 development [39, 40]. A recent study has shown that TGF-*β*, IL-1*β*, and IL-6 are required for Th17 production [41]. In this respect, the VA samples protected mice from sepsis-induced symptoms by inhibiting the synthesis of inflammatory cytokines. Additionally, TGF-*β* appeared to block the activation of lymphocytes and monocyte-derived phagocytes. Downregulation of TGF-*β* for mice pretreated with the VA samples might enhance phagocytic activity of macrophages [42]. 

This study also confirmed that the preparation methods significantly affected the bioactive properties of the VA samples. Our results indicated that the VA powder, dehydrated by lyophilization, might preserve more bioactive components showing a better preventive effect against *S*. *aureus* than the other VA extract samples. Batchelder [13] indicated that therapeutical substances were destroyed in the heating phase of most drying methods. Yudin and Dubryakov [43] also suggested that boiling antler tips destroyed their bioactive potential. Besides heating temperature of extraction and dehydration, the solvents used in extraction of the VA samples also played an important role for preserving bioactive components. The quality of deer antler has often been evaluated by the amount of gangliosides, a sialic acid-containing glycosphingolipid [44]. Ganglioside GM311 and phophatidylcholines with saturated fatty acyl chains [9, 45] might be components related to the proliferation of lymphocytes. Calder et al. [46] also demonstrated the role of fatty acids in the modulation of immune responses. Many other substances from VA such as chondroitin sulfate have also been identified and claimed to contain active components. Chondroitin sulfate, a potent anti-inflammatory agent, is the most prominent glycosaminoglycans in velvet antler tissue. This component has been used by arthritis patients with excellent results. Water extracting methods (such as cold water-soaked extraction and water-boiled extraction) might only extract water soluble bioactive substances from VA (carbohydrates, hexosamines, hydronyprolines, mucopolysacchride, sialic acids, uronic acids, etc.). However, it might be difficult to obtain water insoluble fatty acids (prostaglandins, phospholipids, glycolipids, gangliosides, etc.), which might also possess the therapeutic effects.

## 5. Conclusion

This study has demonstrated the protective mechanisms induced by the VA samples in *S. aureus*-infected animals. The protective mechanisms of the VA samples might include an immune enhancer and a proinflammatory cytokine suppressor. Pretreatment of the VA samples stimulated the proliferation of splenocytes and macrophages enhancing the immune functions. The first protective effect against *S. aureus* given by the VA samples was the upregulated phagocytic activity of monocytes/macrophages. The second protective effect was the reduced synthesis of sepsis-inducing proinflammatory cytokines at a later stage of bacterial infections. Both enhancing phagocytic activity and inhibiting proinflammatory cytokines played important roles for the VA samples in the clearance of *S. aureus* in the infected mice. The VA powder, dehydrated by lyophilization, might preserve more bioactive components and show a better preventive effect against *S. aureus* than the other VA extract samples. This report is thought to be the first to evaluate the effects of velvet antler (VA) of Formosan sambar deer and its extracts on the antiinfective activity against pathogenic *Staphylococcus aureus.*

## Figures and Tables

**Table 1:** Bacterial counts in organs and organ weights of *S. aureus*-infected mice administrated with the different VA samples.

	Bacterial count		Organ weight	
	PLF* (log cfu mL^−1^)	Kidney (log cfu g^−1^)	Kidney (mg)	Spleen (mg)
Water (negative control)	0.0 ± 0.0^c^	0.0 ± 0.0^c^	245 ± 7^c^	152 ± 23^b^
Water (positive control)	4.7 ± 0.9^a^	4.3 ± 0.1^a^	343 ± 50^a^	167 ± 34^a^
VACWS extract	3.8 ± 0.2^b^	4.1 ± 0.7^a^	318 ± 44^ab^	191 ± 32^a^
VAWB extract	3.7 ± 0.5^b^	3.8 ± 0.4^ab^	299 ± 46^ab^	192 ± 60^a^
VA powder (500 mg kg^−1^ mouse body weight)	3.8 ± 0.2^b^	3.1 ± 1.0^b^	272 ± 27^b^	169 ± 20^a^

Values are mean ± S.E.M.

Negative control means mice without i.p. *S. aureus* infection.

^a–c^Means within the same column and the same group without the same superscripts differ significantly (*P* <  .05, *n* = 5).

*PLF: peritoneal lavage fluid.

**Table 2:** Bacterial counts in organs and organ weights of *S. aureus*-infected mice administrated with the different dosages of the VA powder samples.

	Bacterial count		Organ weight	
VA powder (mg kg^−1^ mouse body weight)	PLF* (log cfu mL^−1^)	Kidney (log cfu g^−1^)	Kidney (mg)	Spleen (mg)
Negative control	0.0 ± 0.0^c^	0.0 ± 0.0^c^	245 ± 7^c^	152 ± 23^b^
Positive control	4.4 ± 0.9^a^	6.1 ± 0.8^a^	300 ± 17^a^	198 ± 20^a^
125	4.8 ± 0.8^a^	4.9 ± 1.0^b^	276 ± 19^a^	178 ± 21^a^
250	3.5 ± 0.6^b^	4.7 ± 0.9^b^	262 ± 13^b^	183 ± 25^a^
500	3.9 ± 0.4^b^	4.8 ± 0.6^b^	265 ± 9^b^	176 ± 22^a^

Values are mean ± S.E.M.

^a–c^Means within the same column and the same group without the same superscripts differ significantly (*P* <  .05, *n* = 8).

*PLF: peritoneal lavage fluid.
